# REV1: A novel biomarker and potential therapeutic target for various cancers

**DOI:** 10.3389/fgene.2022.997970

**Published:** 2022-09-29

**Authors:** Ning Zhu, Yingxin Zhao, Mi Mi, Yier Lu, Yinuo Tan, Xuefeng Fang, Shanshan Weng, Ying Yuan

**Affiliations:** ^1^ Department of Medical Oncology, The Second Affiliated Hospital, Zhejiang University School of Medicine, Hangzhou, Zhejiang, China; ^2^ Cancer Institute, Key Laboratory of Cancer Prevention and Intervention, Ministry of Education, The Second Affiliated Hospital, Zhejiang University School of Medicine, Hangzhou, Zhejiang, China; ^3^ Cancer Center, Zhejiang University, Hangzhou, Zhejiang, China

**Keywords:** REV1, translesion synthesis (TLS), prognostic biomarker, drug sensitivity, single nucleotide polymorphism (SNP)

## Abstract

**Background:** REV1 is a member of the translesion synthesis DNA polymerase Y family. It is an essential player in a variety of DNA replication activities, and perform major roles in the production of both spontaneous and DNA damage-induced mutations. This study aimed to explore the role of REV1 as a prognostic biomarker and its potential function regulating the sensitivity of anti-tumor drugs in various cancers.

**Methods:** We analyzed the impact of REV1 gene alterations on patient prognosis and the impact of different REV1 single nucleotide polymorphisms (SNP) on protein structure and function using multiple online prediction servers. REV1 expression was assessed using data from Oncomine, TCGA, and TIMER database. The correlation between REV1 expression and patient prognosis was performed using the PrognoScan and Kaplan-Meier plotter databases. The IC50 values of anti-cancer drugs were downloaded from the Genomics of Drug Sensitivity in Cancer database and the correlation analyses between REV1 expression and each drug pathway’s IC50 value in different tumor types were conducted.

**Results:** Progression free survival was longer in REV1 gene altered group comparing to unaltered group [Median progression free survival (PFS), 107.80 vs. 60.89 months, *p* value = 7.062e-3]. REV1 SNP rs183737771 (F427L) was predicted to be deleterious SNP. REV1 expression differs in different tumour types. Low REV1 expression is associated with better prognosis in colorectal disease specific survival (DSS), disease-free survival (DFS), gastric overall survival (OS), post progression survival (PPS) and ovarian (OS, PPS) cancer while high REV1 expression is associated with better prognosis in lung [OS, relapse free survival (RFS), first progession (FP), PPS] and breast (DSS, RFS) cancer. In colon adenocarcinoma and rectum adenocarcinoma and lung adenocarcinoma, low expression of REV1 may suggest resistance to drugs in certain pathways. Conversely, high expression of REV1 in acute myeloid leukemia, brain lower grade glioma, small cell lung cancer and thyroid carcinoma may indicate resistance to drugs in certain pathways.

**Conclusion:** REV1 plays different roles in different tumor types, drug susceptibility, and related biological events. REV1 expression is significantly correlated with different prognosis in colorectal, ovarian, lung, breast, and gastric cancer. REV1 expression can be used as predictive marker for various drugs of various pathways in different tumors.

## 1 Introduction

Translesion synthesis (TLS) is a DNA damage bypass process involving a group of DNA polymerases including REV1, Pol *η*, *ι*, *κ*, and *ζ*, which together tolerate DNA damage and lead to mutations (Yamanaka et al., 2017). REV1 is a member of the TLS DNA polymerase Y family with dCMP transferase activity that helps bypass certain lesions and functions as a scaffolding protein associated with several TLS DNA polymerases (Gan et al., 2008; Washington et al., 2010). REV1 is an essential player in a variety of DNA replication activities, and perform major roles in the production of both spontaneous and DNA damage-induced mutations (Lawrence, 2002). A number of studies indicate that REV1 has been linked to the development of some cancers (Sakiyama et al., 2005; He et al., 2008; Dumstorf et al., 2009; Xu et al., 2013; Goricar et al., 2014). Studying REV1 will increase our understanding of the origin of cancer, as mutations are an important feature of cancer development.

In our study, we analyzed the impact of REV1 gene alterations on patient outcomes and the impact of different REV1 single nucleotide polymorphisms (SNPs) on protein structure and function using multiple online prediction servers. We also explored the link between the expression of REV1 and cancer patient outcome as well as the correlation between REV1 expression and IC50 of various drugs in different tumor types.

Our study conducted a comprehensive assessment of REV1, explored the role of REV1 in various cancers as a prognostic biomarker and further highlight a potential function whereby REV1 may regulate the sensitivity of tumor cells to specific drugs.

## 2 Materials and methods

### 2.1 Molecular structure of REV1

The summary of molecular structure and function of REV1 were based on the comprehensive literature results. Illustrator for biological sequences (IBS) (http://ibs.biocuckoo.org/) was used to create a schematic of the protein domain (Liu et al., 2015).

### 2.2 REV1 mutation analysis

Copy number amplification (CNA) and mutation status of REV1 in pan-cancer samples were obtained from the cBioPortal database (https://www.cbioportal.org/). We also analyzed the impact of REV1 gene alterations on patient outcomes using cBioPortal database. In addition, REV1 SNPs were searched in NCBI dbSNP database (https://www.ncbi.nlm.nih.gov/snp/), we selected 14 nonsynonymous SNPs (nsSNPs) which have been reported for further analyses. Eight different online servers (SIFT, PolyPhen-2, PANTHER, SNPs&GO, PROVEAN, PredictSNP, Mutation Taster2, and Mutation assessor) based on multiple algorithms (Supplementary Table S1), were used for structural and functional annotation. Stability analysis was carried out using I-Mutant2.0, MUpro, and iStable (Supplementary Table S1). We performed 3D modeling analysis of the wild-type and mutant UmuC domains of REV1 protein.

### 2.3 REV1 expression analysis

We assessed the expression of REV1 in multiple tumour and normal tissue types using the Oncomine database. We also analyzed immunohistochemistry staining images from the Human Protein Atlas (HPA) website (https://www.proteinatlas.org/) to study the protein expression of REV1 in multiple tumour and normal tissue types. We further used the TCGA and TIMER databases to assess how REV1 expression differs in particular tumour types. We also explored the link between the expression of REV1 and cancer patient outcome using the PrognoScan and Kaplan-Meier plotter databases (Supplementary Table S1).

### 2.4 Drug sensitivity analysis

The IC50 values of drugs, each drug corresponding signaling pathway and gene expression profiles in the relative cell lines (E-MTAB3610) were downloaded from the Genomics of Drug Sensitivity in Cancer (GDSC) database (https://www.cancerrxgene.org/) and ArrayExpress database (https://www.ebi.ac.uk/arrayexpress/). The correlation analyses between REV1 expression and each drug pathway’s IC50 value in different tumor types were conducted.

### 2.5 Statistical analysis

Statistical analyses were conducted using R software (version 3.6.2) and attached packages. Survival analyses were performed using the log-rank test, the Kaplan-Meier method, and the Cox regression model. Correlation analyses were conducted using Pearson’s test. Two-tailed *p* value < 0.05 was considered as statistically significant. *p*-value Significant Codes: 0 ≤ *** < 0.001 ≤ ** < 0.01 ≤ * < 0.05 ≤ < 0.1.

## 3 Results

### 3.1 Molecular structure and function of REV1

**FIGURE 1:**
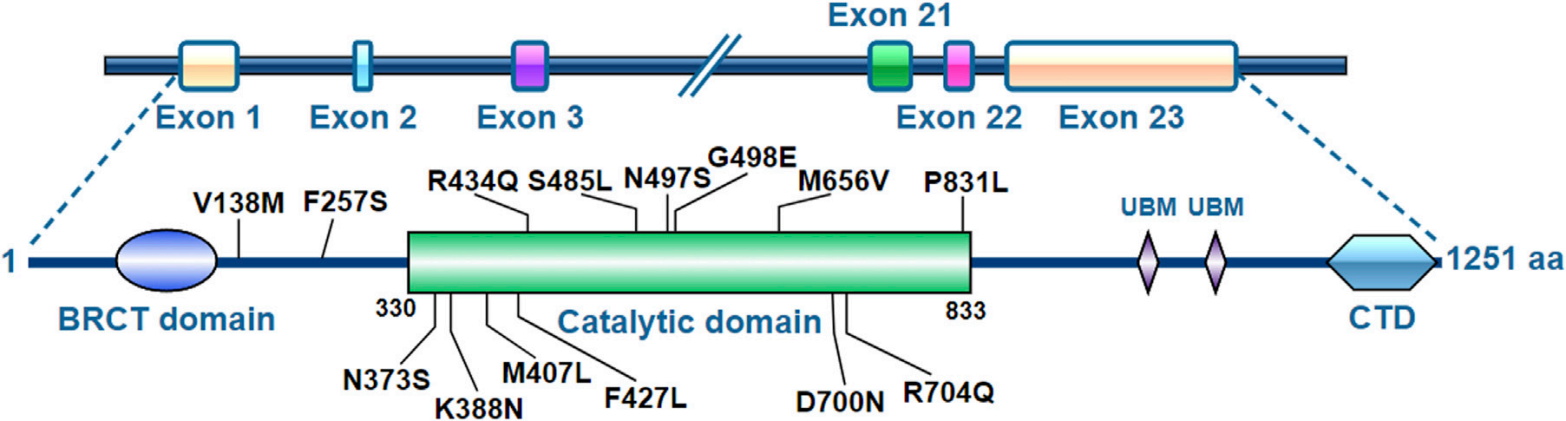
Molecular structure of REV1.

The human REV1 gene locates at chromosome 2q11.2 and has 23 exons encoding the REV1 protein consisting of 1,251 amino acids (Figure 1). The middle part of the REV1 protein (amino acids 330−833) is the catalytic domain, while the C- and N-terminal regions contain protein-protein interaction domains [e.g., ubiquitin-binding motif (UBM), C-terminal domain (CTD) and BRCT] (Swan et al., 2009; Sale, 2013).

REV1 is a member of the eukaryotic Y-polymerase family of TLS DNA polymerases (Murakumo et al., 2001; Guo et al., 2003; Ohashi et al., 2004; Tissier et al., 2004). It plays a central role in TLS by participating in protein-protein interactions through two distinct interfaces at its CTD. It recruits the TLS polymerases POL *κ*, POL *ι*, and POL *η* using one interface, and recruits POL *ζ* through interaction with REV7 components by another interface (Pozhidaeva et al., 2012; Wojtaszek et al., 2012; Yamanaka et al., 2017). In addition to this non-catalytic role, REV1, unique among polymerases, has its own catalytic activity as a deoxycytidyl transferase, utilizing a protein-template directed mechanism (Nair et al., 2005; Swan et al., 2009).

### 3.2 The role of REV1 in carcinogenesis and prognosis

#### 3.2.1 REV1 single nucleotide polymorphisms in various cancers

**FIGURE 2:**
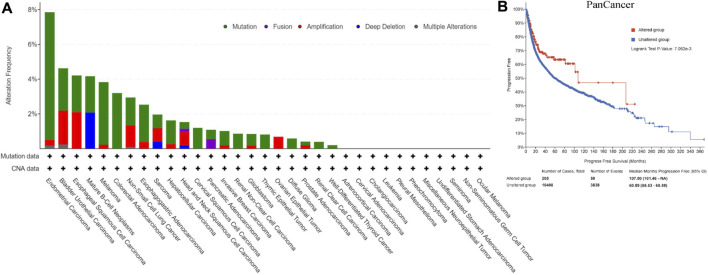
Copy number amplification and mutation status of REV1 **(A)** and corresponding progression free survival **(B)** in pan-cancer samples.

Figure 2A showed CNA and mutation status of REV1 in pan-cancer samples from cBioPortal database. Survival analysis of REV1 gene alterations suggested that progression free survival (PFS) was longer in REV1 gene altered group comparing to unaltered group (Median PFS, 107.80 vs. 60.89 months, *p* value = 7.062e-3) (Figure 2B). However, the disease specific survival (DSS) and disease-free survival (DFS) of the two groups were not statistically different based on data from cBioPortal database.

There were 23,494 REV1 SNPs in NCBI dbSNP database, 19,933 SNPs in intronic region, 1,183 SNPs in 5′UTR region, 432 SNPs in 3′UTR region, and 926 SNPs were nsSNPs and 422 SNPs were synonymous SNPs in coding sequence. We selected 14 nsSNPs which have been reported for further analyses. Out of 14 nsSNPs, only rs183737771 (F427L) was predicted to be deleterious SNPs in all computational algorithms (Table 1). Nine nsSNPs were predicted to decrease protein stability, meanwhile, one nsSNP was predicted to increase (Table 1).

**TABLE 1:** High risk nsSNPs identified in silico programs and effect of nsSNPs on protein stability predicted by silico programs.

SNP ID	AA change	Structural and functional annotation								Protein stability		
		SIFT	Polyphen2	PANTHER	SNPs&Go	PROVEAN	PredictSNP	Mutation Taster2	Mutation assessor	i-Mutant2.0	Mupro	iStable
rs3087399	N373S	Tolerated	Benign	Possibly damaging	Neutral	Deleterious	Neutral	Benign	Medium	Decrease	Increase	Increase
rs3087386	F257S	Tolerated	Benign	Possibly damaging	Neutral	Neutral	Neutral	Benign	Neutral	Decrease	Decrease	Decrease
rs3087403	V138M	Tolerated	Benign	Possibly damaging	Neutral	Neutral	Neutral	Benign	Low	Decrease	Decrease	Decrease
rs72550807	K388N	Deleterious	Probably Damaging	Possibly damaging	Neutral	Deleterious	Neutral	Benign	Medium	Decrease	Decrease	Decrease
rs138841507	S485L	Tolerated	Benign	Possibly damaging	Neutral	Neutral	Neutral	Benign	Neutral	Increase	Increase	Increase
rs144046214	G498E	Deleterious	Probably Damaging	Possibly damaging	Neutral	Deleterious	Deleterious	Benign	Medium	Decrease	Increase	Increase
rs183737771	F427L	Deleterious	Probably Damaging	Possibly damaging	Disease	Deleterious	Deleterious	Deleterious	High	Decrease	Decrease	Decrease
rs148052685	R434Q	Deleterious	Probably Damaging	Possibly damaging	Neutral	Deleterious	Deleterious	Deleterious	Low	Decrease	Decrease	Decrease
rs3087394	M656V	Deleterious	Benign	Possibly damaging	Neutral	Neutral	Neutral	Benign	Medium	Decrease	Decrease	Decrease
rs28382941	D700N	Deleterious	Benign	Possibly damaging	Neutral	Deleterious	Neutral	Benign	Medium	Decrease	Decrease	Decrease
rs28382942	R704Q	Tolerated	Benign	Possibly damaging	Neutral	Neutral	Neutral	Benign	Neutral	Decrease	Decrease	Decrease
rs139685542	P831L	Tolerated	Benign	Possibly damaging	Neutral	Neutral	Neutral	Benign	Neutral	Decrease	Increase	Increase
rs200184935	M407L	Tolerated	Benign	Possibly damaging	Neutral	Neutral	Neutral	Benign	Low	Decrease	Increase	Increase
rs180712764	N497S	Tolerated	Probably Damaging	Possibly damaging	Neutral	Neutral	Neutral	Benign	Neutral	Decrease	Increase	Increase

**FIGURE 3:**
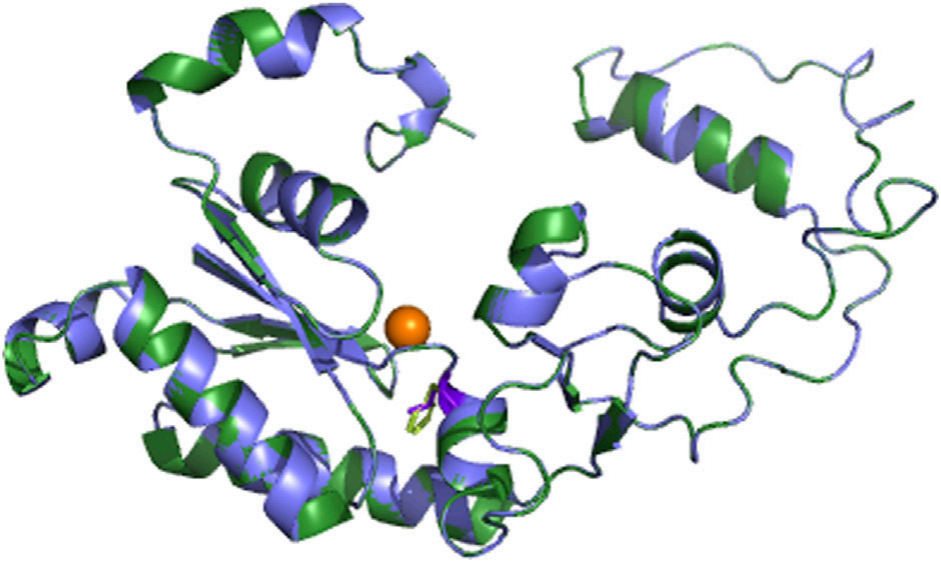
3D models of UmuC domain.

**FIGURE 4:**
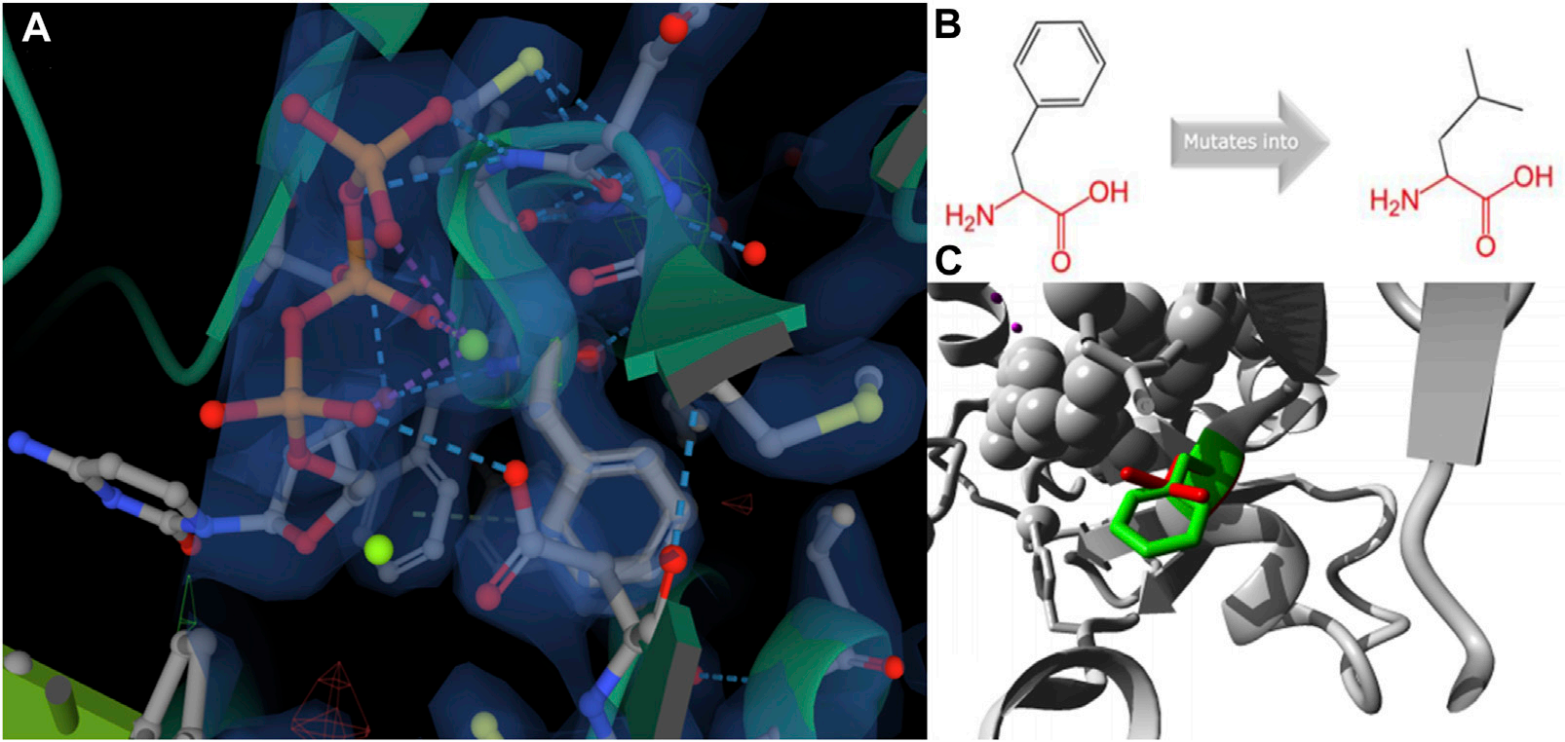
3D structure **(A, C)** and chemical structure changes **(B)** of position 427 on REV1 protein.

To further analyze why F427L is a deleterious SNP, we performed 3D model predictions. We selected 3gqc.1. A as template in 3D modeling analysis (Figure 3). We found that compared with wild-type domain, F427L has no significant effect on protein structure. However, the wild-type 3D structure (3GQC) showed that position 427 is involved in the contact of Mg^2+^ and dCTP (Figure 4A). Project HOPE analysis revealed that after the phenylalanine at position 427 is mutated to leucine, the mutated residue is smaller than that of the wild-type residue, then creates a blank space in the protein core (Figures 4B,C), which probably interferes with the interaction with Mg^2+^ and leads to the loss of interaction with dCTP, thereby affecting protein function (Venselaar et al., 2010).

#### 3.2.2 REV1 expression in different cancer and normal tissues

**FIGURE 5:**
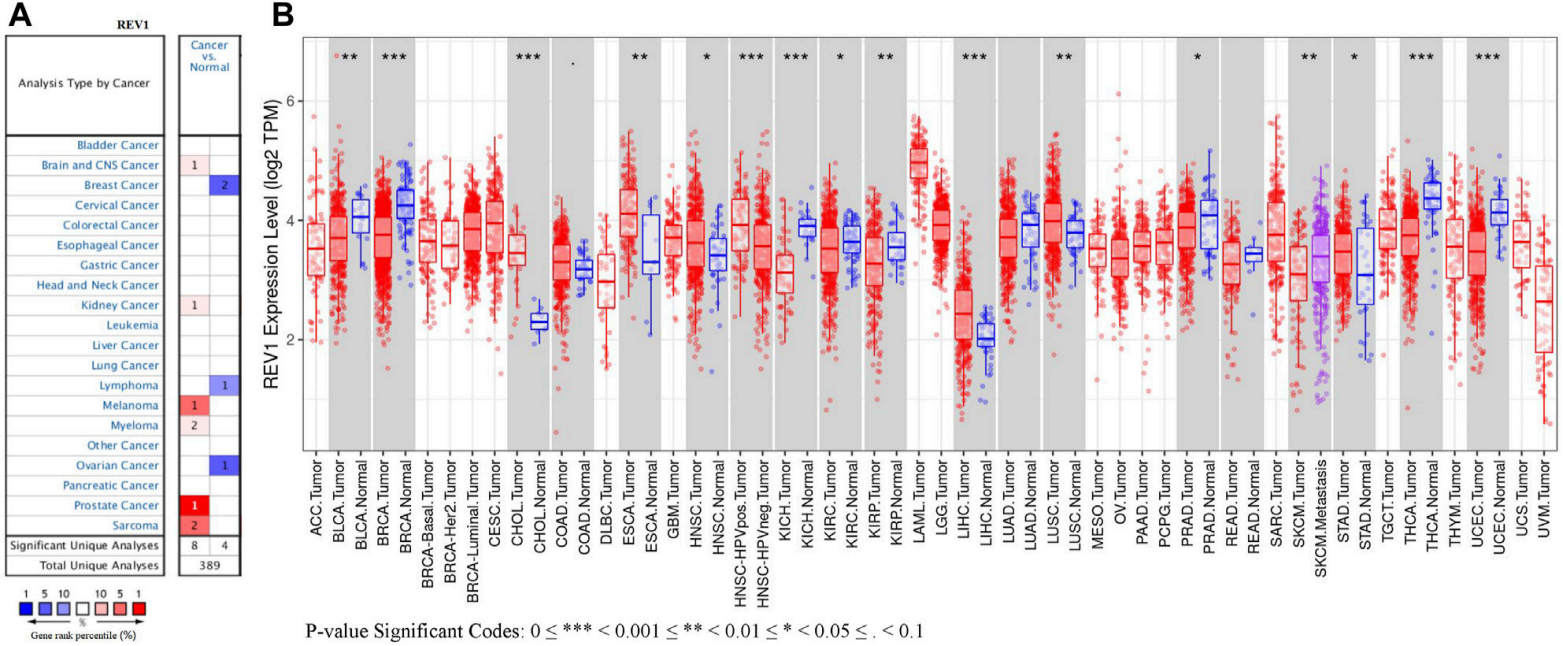
The expression level of REV1 in different types of tumor tissues and normal tissues. **(A)** Oncomine database. **(B)** TIMER database.

The expression of REV1 in multiple tumour and normal tissue types from the Oncomine database revealed that expression of this gene was elevated relative to normal tissue controls for brain, renal, prostate cancers, melanoma, myeloma and sarcoma. We also found that relative to normal tissue controls, REV1 expression was lower in breast, ovarian cancer and lymphoma tissues (Figure 5A). Detailed findings in particular tumour types are showed in Supplementary Table S2.

REV1 expression in tumor tissue samples and normal tissue samples from TCGA and TIMER databases were assessed to figure out how REV1 expression differs in particular tumour types. The results suggested that the expression of REV1 was significantly elevated relative to normal controls in cholangiocarcinoma (CHOL), esophageal carcinoma (ESCA), head and neck cancer (HNSC), liver hepatocellular carcinoma (LIHC), lung squamous cell carcinoma (LUSC) and stomach adenocarcinoma (STAD). In contrast, low REV1 expression was observed in eight cancer types, namely, bladder urothelial carcinoma (BLCA), breast invasive carcinoma (BRCA), kidney chromophobe (KICH), kidney renal clear cell carcinoma (KIRC), kidney renal papillary cell carcinoma (KIRP), prostate adenocarcinoma (PRAD), thyroid carcinoma (THCA) and uterine corpus endometrial carcinoma (UCEC). Differences between the expression of REV1 in tumors and normal adjacent tissue samples in the TCGA data set are shown in Figure 5B.

**FIGURE 6:**
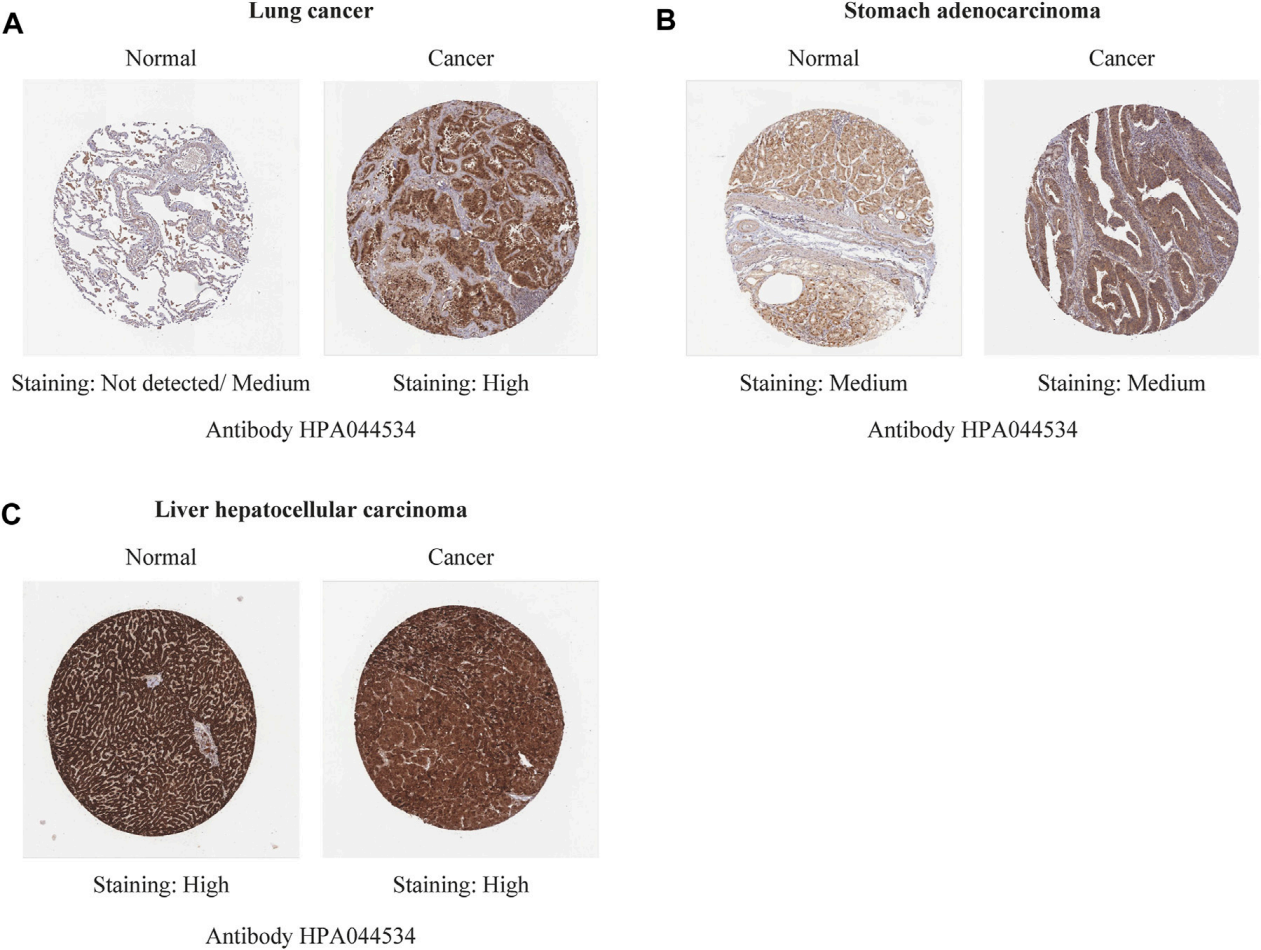
The immunohistochemistry staining images of REV1 in different types of tumor tissues and normal tissues [lung **(A)**, stomach **(B)** and liver **(C)**].

**FIGURE 7:**
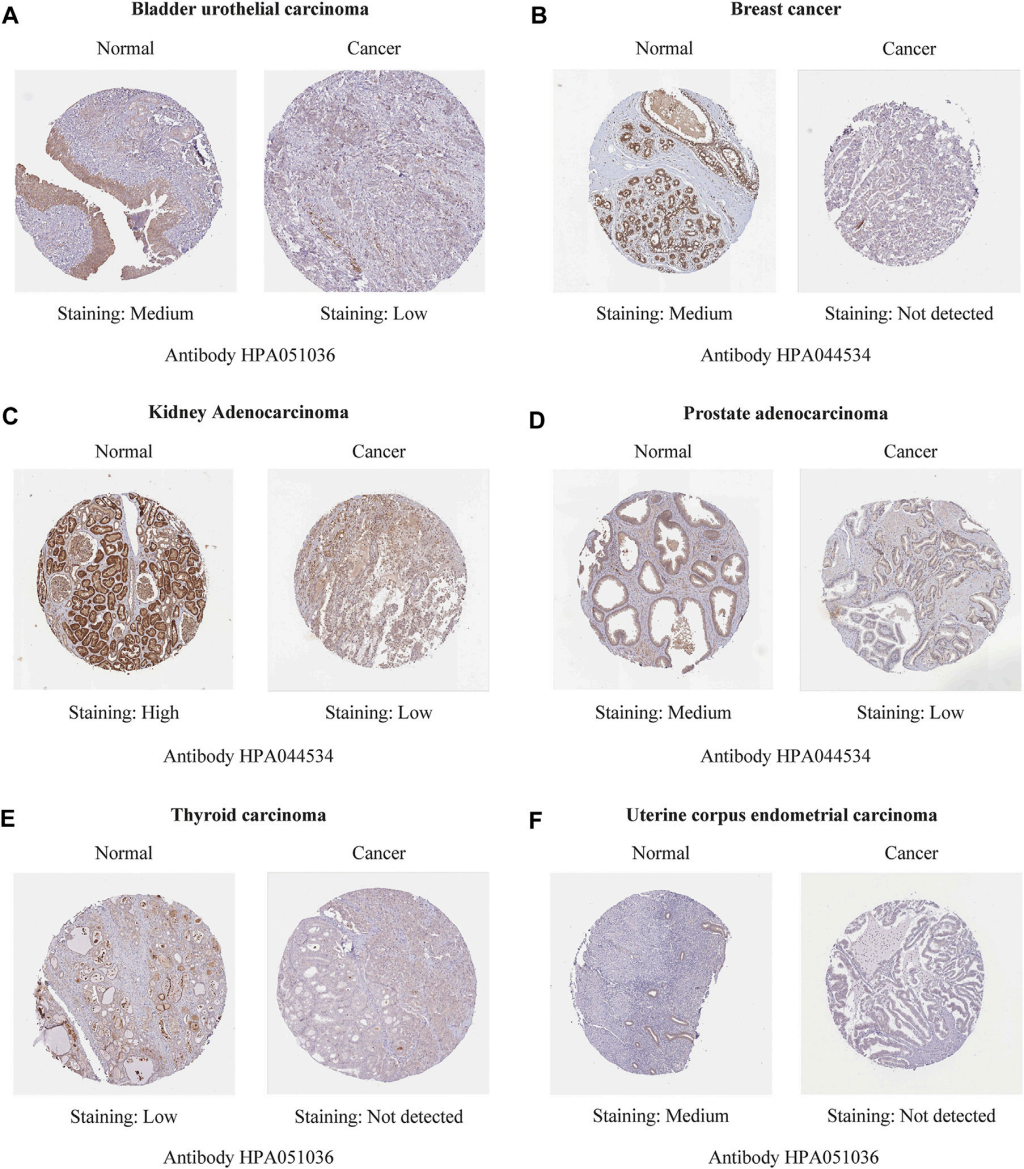
The immunohistochemistry staining images of REV1 in different types of tumor tissues and normal tissues [urinary bladder **(A)**, breast **(B)**, kidney **(C)**, prostate **(D)**, thyroid **(E)**, and endometrium **(F)**].

Immunohistochemistry staining analysis from HPA yielded similar results (Figures 6, 7). REV1 proteins were not expressed or medium expressed in normal lung tissues, while high protein expressions of REV1 were expressed in lung cancer tissues (Figure 6A). Medium protein expressions of REV1 were observed in urinary bladder, breast, prostate and endometrium normal tissues, while not detected (breast cancer, endometrial carcinoma) or low (urothelial carcinoma, prostate adenocarcinoma) protein expressions of REV1 were observed in corresponding cancer tissues (Figures 7A,B,D,F). REV1 proteins were high expressed in normal kidney tissues, while low protein expressions of REV1 were expressed in kidney adenocarcinoma tissues (Figure 7C).

#### 3.2.3 The association between REV1 expression and patient prognosis

**FIGURE 8:**
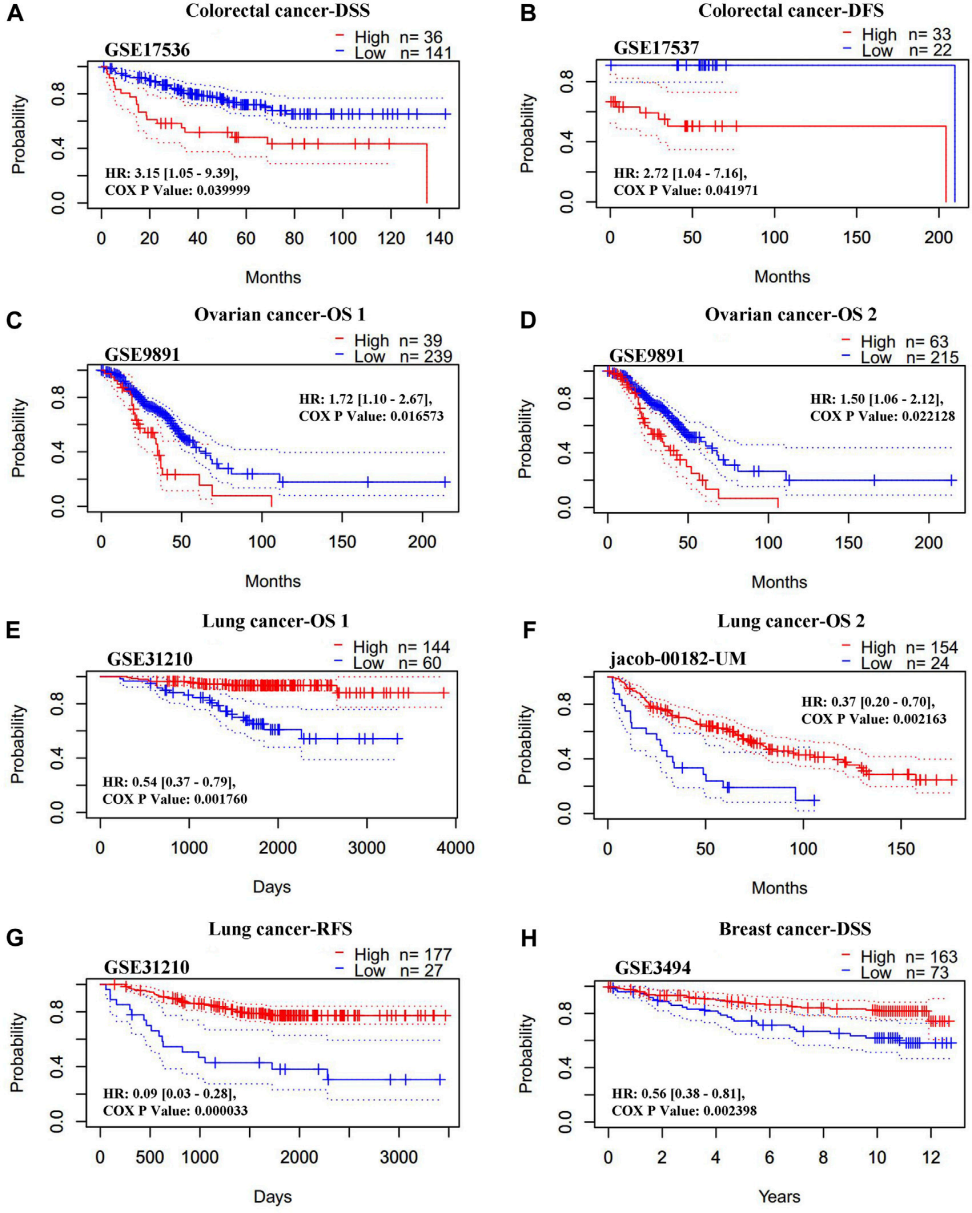
Correlation between REV1 expression and prognosis of various types of cancer [colorectal cancer **(A, B),** ovarian cancer **(C, D)**, lung cancer **(E–G)**, and breast cancer **(H)**] (PrognoScan database).

The association between the REV1 expression and patient prognosis were analyzed using the PrognoScan database (Supplementary Tables S3–S7). We found that high REV1 expression is associated with poorer prognosis in colorectal (DSS: HR, 3.15, 95% CI, 1.05–9.39, *p* = 0.039999; DFS: HR, 2.72, 95% CI, 1.04–7.16, *p* = 0.041971) and ovarian cancer (overall survival (OS) 1: HR, 1.72, 95% CI, 1.10–2.67, *p* = 0.016573; OS 2: HR, 1.50, 95% CI, 1.06–2.12, *p* = 0.022128) (Figures 8A–D). However, as shown in Figures 8E–H, survival curves identified that high expression of REV1 indicated better prognosis in lung [OS 1: HR, 0.54, 95% CI, 0.37–0.79, *p* = 0.001760; OS 2: HR, 0.37, 95% CI, 0.20–0.70, *p* = 0.002163; relapse free survival (RFS): HR, 0.09, 95% CI, 0.03–0.28, *p* = 0.000033] and breast cancer (DSS: HR, 0.56, 95% CI, 0.38–0.81, *p* = 0.002398).

**FIGURE 9:**
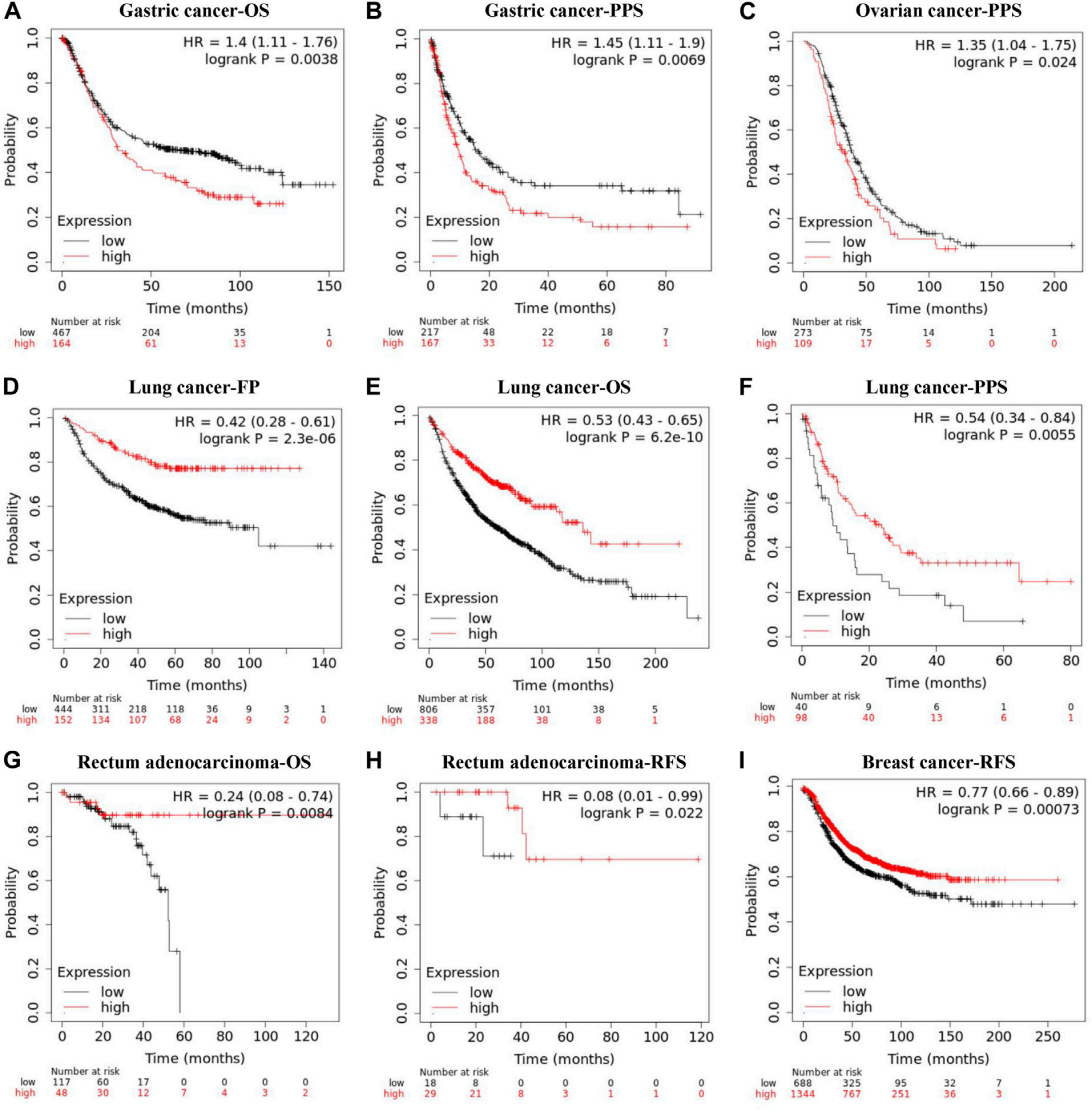
Correlation between REV1 expression and prognosis of various types of cancer [gastric cancer **(A, B)**, ovarian cancer **(C)**, lung cancer **(D–F)**, rectum adenocarcinoma **(G, H)**, and breast cancer **(I)**] (Kaplan-Meier Plotter database).

The Kaplan-Meier plotter database was also employed to assess the relationship between REV1 expression and patient prognosis in a range of cancer types. As shown in Figures 9A–C, the elevation expression of REV1 to be significantly linked with a poorer prognosis in gastric cancer [OS: HR, 1.4, 95% CI, 1.11–1.76, *p* = 0.0038; post progression survival (PPS): HR, 1.45, 95% CI, 1.11–1.9, *p* = 0.0069] and ovarian cancer (PPS: HR, 1.35, 95% CI, 1.04–1.75, *p* = 0.024). However, we found reduced REV1 expression to be correlated with poorer patient prognosis in lung cancer (first progession (FP): HR, 0.42, 95% CI, 0.28–0.61, *p* = 2.3e-06; OS: HR, 0.53, 95% CI, 0.43–0.65, *p* = 6.2e-10; PPS: HR, 0.54, 95% CI, 0.34–0.84, *p* = 0.0055) (Figures 9D–F), Rectum adenocarcinoma (OS: HR, 0.24, 95% CI, 0.08–0.74, *p* = 0.0084; RFS: HR, 0.08, 95% CI, 0.01–0.99, *p* = 0.022) (Figures 9G,H) and breast cancer (RFS: HR, 0.77, 95% CI, 0.66–0.89, *p* = 0.00073) (Figure 9I). There was no statistically significant relationship between the expression of REV1 and the prognosis of breast cancer patients [distant metastasis-free survival (DMFS), OS and PPS], gastric cancer (FP) and ovarian cancer (OS and PFS) (Supplementary Figure S1).

### 3.3 The role of REV1 in sensitivity of cancer treatment

#### 3.3.1 The association between REV1 expression and cancer treatment sensitivity

**FIGURE 10:**
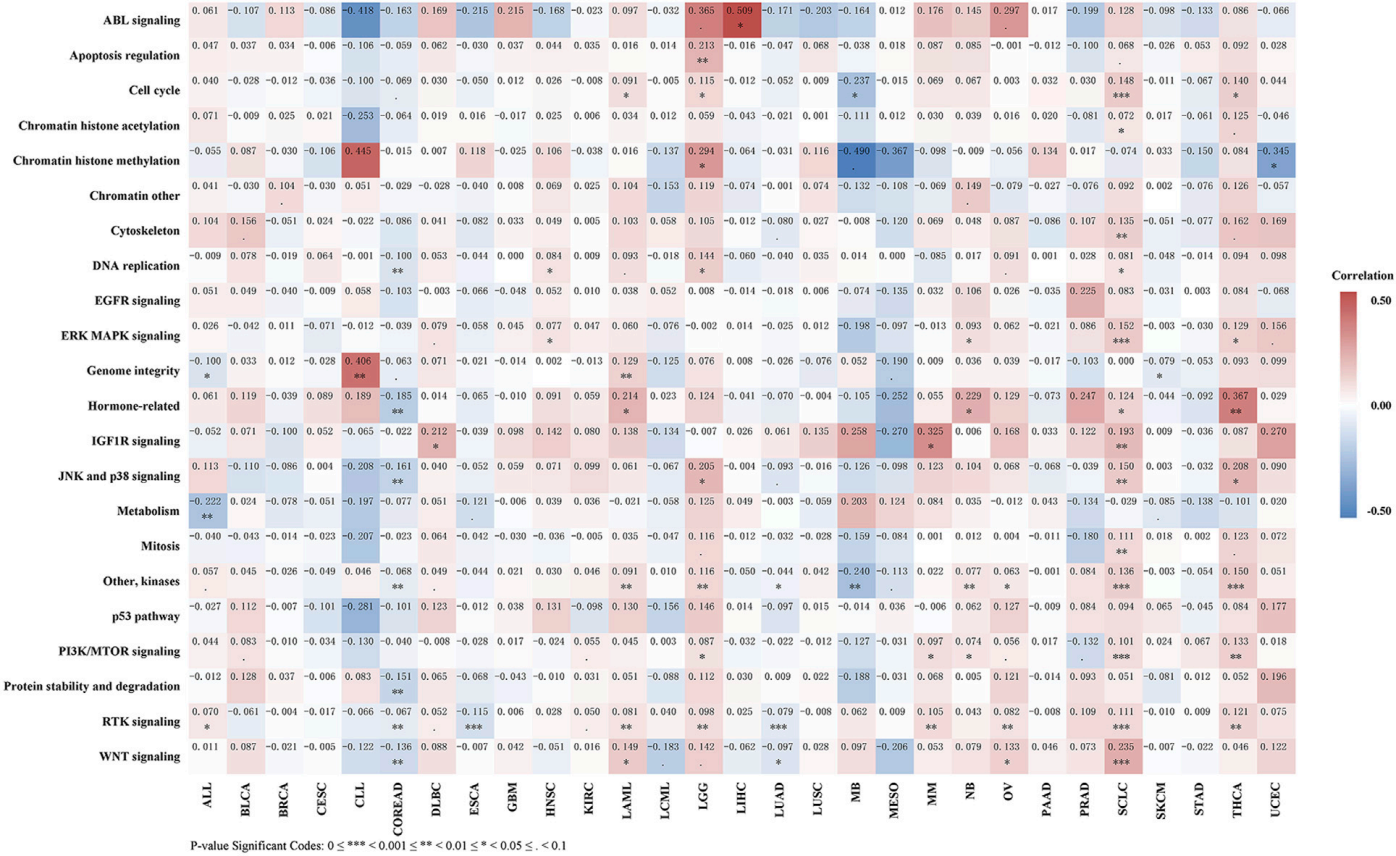
Correlation heatmap of REV1 expression and drug pathway’s IC50 in different tumors.

As shown in Figure 10 and Table S8, REV1 expression is negatively correlated with IC50 of drugs of DNA replication, hormone-related, JNK and p38 signaling, kinases, protein stability and degradation, RTK signaling, and WNT signaling pathways in colon adenocarcinoma and rectum adenocarcinoma and drugs of kinases, RTK signaling, and WNT signaling pathways in lung adenocarcinoma. In contrast, in acute myeloid leukemia, brain lower grade glioma, small cell lung cancer and thyroid carcinoma, REV1 expression is positively correlated with IC50 of drugs of various pathways (acute myeloid leukemia: Cell cycle, Genome integrity, Hormone-related, kinases, RTK signaling, and WNT signaling pathways; brain lower grade glioma: Cell cycle, Chromatin histone methylation, DNA replication, JNK and p38 signaling, kinases, PI3K/MTOR signaling, and RTK signaling pathways; small cell lung cancer: Cell cycle, Chromatin histone acetylation, Cytoskeleton, DNA replication, ERK MAPK signaling, Hormone-related, IGF1R signaling, JNK and p38 signaling, Mitosis, kinases, PI3K/MTOR signaling, RTK signaling, and WNT signaling pathways; thyroid carcinoma: Cell cycle, ERK MAPK signaling, Hormone-related, JNK and p38 signaling, kinases, PI3K/MTOR signaling, and RTK signaling pathways). In acute lymphoblastic leukemia, REV1 expression is negatively correlated with IC50 of drugs of Genome integrity and metabolism pathways while positively correlated with IC50 of drugs of RTK signaling pathway. Thus, REV1 expression can be used as predictive marker for various drugs of various pathways in different tumors.

## 4 Discussion

REV1 is one of the key proteins in TLS. TLS is a DNA damage tolerance process, which contributes to cell survival by bypass of the unrepaired DNA lesions. TLS functions in an error-prone manner and sometimes can actively promote the generation of mutations (Waters et al., 2009). These accumulated errors in the DNA may play a key role in the initiation of various types of cancers (Schoket, 2004). REV1 also plays important role in replication stress response (RSR). The RSR is activated in response to DNA lesions or intrinsic replication fork barriers. Replication forks stalled at DNA lesions can restart replication by firing dormant origins, repriming replication, reversing the stalled fork or activating the DNA damage tolerance pathways (Zeman and Cimprich, 2014). ssDNA gaps are frequent structures that accumulate on newly synthesized DNA under conditions of replication stress (Quinet et al., 2014). Tirman et al. (2021) showed that REV1-POLζ-mediated trans-DNA damage synthesis promotes ssDNA gap filling in G2 and S, affecting cell survival and genome stability. Similar findings were found by Nayak et al. (2020), Moreover, Yang et al. (2015) found that REV1 protects nascent replication tracts intact by stabilizing RAD51 filaments, to block nascent replication tracts from degradation in response to replication stress.

Studies have shown that REV1 may play an oncogenic role in lung and intestinal tumor. Overexpression of REV1 promotes the development of carcinogen-induced intestinal adenomas *via* accumulation of point mutation and suppression of apoptosis proportionally to the REV1 expression level (Sasatani et al., 2017). And an animal experiment showed that in 27% of the carcinogen-exposed mice, REV1 inhibition completely abolished tumor formation (Dumstorf et al., 2009). We assessed REV1 expression using tumor tissue data and normal tissue data from TCGA and TIMER databases. We found that REV1 was overexpressed in six cancer types, namely, CHOL, ESCA, HNSC, LIHC, LUSC, and STAD. In contrast, low REV1 expression was observed in only eight cancer types, namely, BLCA, BRCA, KICH, KIRC, KIRP, PRAD, THCA, and UCEC. It can be seen that REV1 has different effects on the occurrence and development of tumors in different tumor types.

In our studies, we also focused on the role of REV1 in various cancers as a prognostic biomarker and the potential function of REV1 regulating the sensitivity of tumor cells to specific drugs. These findings may provide a certain reference for the development of REV1-targeted therapeutics.

REV1 as a potential prognostic biomarker, our research found REV1 gene alterations are related to PFS prognosis in pan-cancer samples while nsSNP F427L is predicted to be deleterious SNP. That means different REV1 SNPs play different roles in different tumor types, drug susceptibility, and related biological events. According to current literature, Yeom et al. (2016) divided the 12 germline variants of REV1 into three types, the “wild-type-like” variants (K388N, S485L, and G498E), the hypoactive variants (F427L, R434Q, M656V, D700N, R704Q, and P831L) and the hyperactive variants (N373S, M407L, and N497S). These mutations may lead to different mutant phenotype and susceptibility to certain chemical and viral carcinogens in affected individuals (Yeom et al., 2016). REV1 N373S SNP was associated with an increased risk of cervical cancer, while F257S SNP is associated with a reduced risk of cervical cancer and an increased risk of lung cancer in people with heavy smoking (Sakiyama et al., 2005; He et al., 2008; Dumstorf et al., 2009; Xu et al., 2013). For REV1 SNP in non-coding region, rs6761390, rs3792142, and rs3792136 have been reported in literature. Specifically, rs6761390 locates at promoter, a putative transcription factor binding site; rs3792142 is a tag SNP located at intron 5 and rs3792136 was located in the intron region (Xu et al., 2013). Minor allele carriers of two REV1 SNPs (rs6761390 and rs3792142) had significantly more often large tumours and tumours with high histological grade and stage than the common homozygotes (Varadi et al., 2011). The heterozygote of REV1 rs3087386 (F257S) and rs3792136 were independent prognostic factors for lung cancer survival with hazard radio (HR) 1.54 (95% CI: 1.12–2.12) and 1.44 (95% CI: 1.06–1.97) respectively (Xu et al., 2013).

As for the correlation between REV1 expression and prognosis, our study suggests that low REV1 expression is associated with better prognosis in colorectal (DSS, DFS), gastric (OS, PPS) and ovarian (OS, PPS) cancer while high REV1 expression is associated with better prognosis in lung (OS, RFS, FP, PPS) and breast (DSS, RFS) cancer. These indicated that the functions of REV1 are different or even opposite in different tumors.

REV1 as a potential predictor of drug sensitivity, our study shows that in different tumor types, the expression of REV1 is correlated with the sensitivity of drugs in different mechanisms of pathways. In colon adenocarcinoma and rectum adenocarcinoma and lung adenocarcinoma, low expression of REV1 may suggest resistance to drugs in certain pathways. Conversely, high expression of REV1 in acute myeloid leukemia, brain lower grade glioma, small cell lung cancer and thyroid carcinoma may indicate resistance to drugs in certain pathways.

Currently, there is some evidence that inhibition of TLS polymerase including REV1 not only sensitizes tumor cells to chemotherapeutic drugs, but also reduces the acquisition of drug-induced mutations associated with tumor resistance (Xie et al., 2010). Therefore, TLS inhibition may have dual anticancer effects, and inhibition of TLS polymerase is a promising new approach to improve cancer therapy (Yamanaka et al., 2017).

In recent years, many scientists devoted to develop the small molecule inhibitors targeting REV1 and achieved certain results. In 2017, Sail et al. (2017) identified the first small molecules that exhibit anti-TLS activity in human cancer cells through disruption of the protein-protein interactions between the C-terminal domain of REV1 and the REV1-interacting region. In 2018, Vanarotti et al. (2018) identified the first small-molecule compound, MLAF50, that inhibited the interaction of REV1 UBM2 with ubiquitin through directly binding to REV1 UBM2. In 2019, Wojtaszek et al. (2019) discovered a small molecule inhibitor, JH-RE-06, targeting a nearly featureless surface of REV1 that interacts with the REV7 subunit of POL *ζ*. Binding of JH-RE-06 induces REV1 dimerization, which blocks the REV1-REV7 interaction and POL *ζ* recruitment. Recently, Pernicone et al. (2022) discovered two small molecules (ZINC97017995 and ZINC25496030) from the ZINC12 subset Library that disrupt the assembly of MAD2L2-Rev1 and the formation of an active TLS complex. The above studies all show that these small molecules combined with cisplatin could enhance the sensitivity of human cancer cells to cisplatin while have minimal toxicity on their own. However, the current researches on these small molecule inhibitors are limited to *in vitro* cell experiments and a small number of mouse subcutaneous tumor drug experiments, and clinical trials have not yet been carried out.

In addition, there are many literatures suggesting that REV1 SNP or high level of the REV1 expression is associated with resistance to certain drugs. Goricar et al. (2014), Goricar et al. (2015) reported that the V138M SNP of REV1 gene is associated with poor response to cisplatin chemotherapy in patients with malignant mesothelioma and osteosarcoma. The study also revealed that REV1 gene SNPs were associated with the hematological toxicity of cisplatin in malignant mesothelioma. The REV1 allele V138M SNP (rs3087403) is associated with an increased risk of leukopenia and neutropenia. In contrast, patients with at least one REV1 allele of the F257S SNP (rs3087386) had a reduced risk of neutropenia compared with patients with two wild-type alleles (Goricar et al., 2014). Other researchers have demonstrated *in vivo* experiments that REV1 plays a key role in the development of acquired cyclophosphamide resistance (Xie et al., 2010). In addition, studies have shown that reducing the expression level of REV1 can promote the sensitivity of cells to cisplatin and PARP inhibition (Wang et al., 2012). And high levels of REV1 expression led to resistance of ovarian cancer cells to cisplatin. This may be related to the fact that REV1 can enhance cell survival and the generation of drug-resistant variants in surviving populations (Okuda et al., 2005; Lin et al., 2006). However, recently, studies by Kanayo E. Ikeh et al. (2021) suggested that REV1 inhibition may provide cytoprotection by inducing autophagy during radiation therapy. Thus, REV1 may be an important biomarker in tumor treatment. On the one hand, its decreased level during chemotherapy such as cisplatin and cyclophosphamide will be an indicator of a good response of patients to treatment. On the other hand, patients receiving radiation therapy need to increase REV1 levels.

The results of the TLS process can be error-free or error-prone, depending on the type of DNA damage and the specific polymerase. Therefore, REV1 may be protective or mutagenic for each specific lesion during DNA damage-induced cellular mutagenesis (Yeom et al., 2016). That also explains that the effects of REV1 on the occurrence, development, prognosis and drug sensitivity of tumors vary greatly among different tumor types and different SNPs, which reminds us that REV1, as a novel biomarker and potential therapeutic target, requires specific analysis of specific problems. Our study conducted a comprehensive assessment of REV1, to a certain extent, which may provide some hints in this regard.

## 5 Conclusion

In conclusion, REV1 plays different roles in different tumor types, drug susceptibility, and related biological events. REV1 gene alterations are related to PFS prognosis and nsSNP F427L is predicted to be deleterious SNP. REV1 expression is significantly correlated with different prognosis in colorectal, ovarian, lung, breast, and gastric cancer. REV1 expression can be used as predictive marker for various drugs of various pathways in different tumors.

## Data Availability

Publicly available datasets were analyzed in this study. The names of the repository/repositories and accession number(s) can be found in the article/Supplementary Material.

## Abbreviations

TLS, Translesion synthesis; SNPs, single nucleotide polymorphisms; IBS, Illustrator for biological sequences; CNA, Copy number amplification; nsSNPs, nonsynonymous SNPs; GDSC, Genomics of Drug Sensitivity in Cancer; UBM, ubiquitin-binding motif; CTD, C-terminal domain; DSS, disease specific survival; DFS, disease-free survival; CHOL, cholangiocarcinoma; ESCA, esophageal carcinoma; HNSC, head and neck cancer; LIHC, liver hepatocellular carcinoma; LUSC, lung squamous cell carcinoma; STAD, stomach adenocarcinoma; BLCA, bladder urothelial carcinoma; BRCA, breast invasive carcinoma; KICH, kidney chromophobe; KIRC, kidney renal clear cell carcinoma; KIRP, kidney renal papillary cell carcinoma; PRAD, prostate adenocarcinoma; THCA, thyroid carcinoma; UCEC, uterine corpus endometrial carcinoma; PPS, post progression survival; FP, first progession; DMFS, distant metastasis-free survival; HR, hazard radio
