# Supplementary material for: REV1: A novel biomarker and potential therapeutic target for various cancers
Source: Front Genet. 2022 Sep 29;13:997970. doi: 10.3389/fgene.2022.997970 (PMC9560673; doi:10.3389/fgene.2022.997970)
Supplement: Supplementary file 1 [file DataSheet1.PDF]

Supplementary materials

Content:

Figure S1. No correlation between REV1 expression and prognosis of various types of cancer (Kaplan-Meier Plotter database).

Table S1. Information of in silico algorithms.

Table S2. REV1 expression in cancers vs. normal tissue in oncomine database.

Table S3. Relation between REV1 expression and patient prognosis of different cancer (Disease Free Survival) in Prognoscan database.

Table S4. Relation between REV1 expression and patient prognosis of different cancer (Disease Specific Survival) in Prognoscan database.

Table S5. Relation between REV1 expression and patient prognosis of different cancer (Distant Metastasis Free Survival) in Prognoscan database.

Table S6. Relation between REV1 expression and patient prognosis of different cancer (Overall Survival) in Prognoscan database.

Table S7. Relation between REV1 expression and patient prognosis of different cancer (Relapse Free Survival) in Prognoscan database.

Table S8. The correlation between REV1 expression and drug pathway's IC50 value in different tumors.

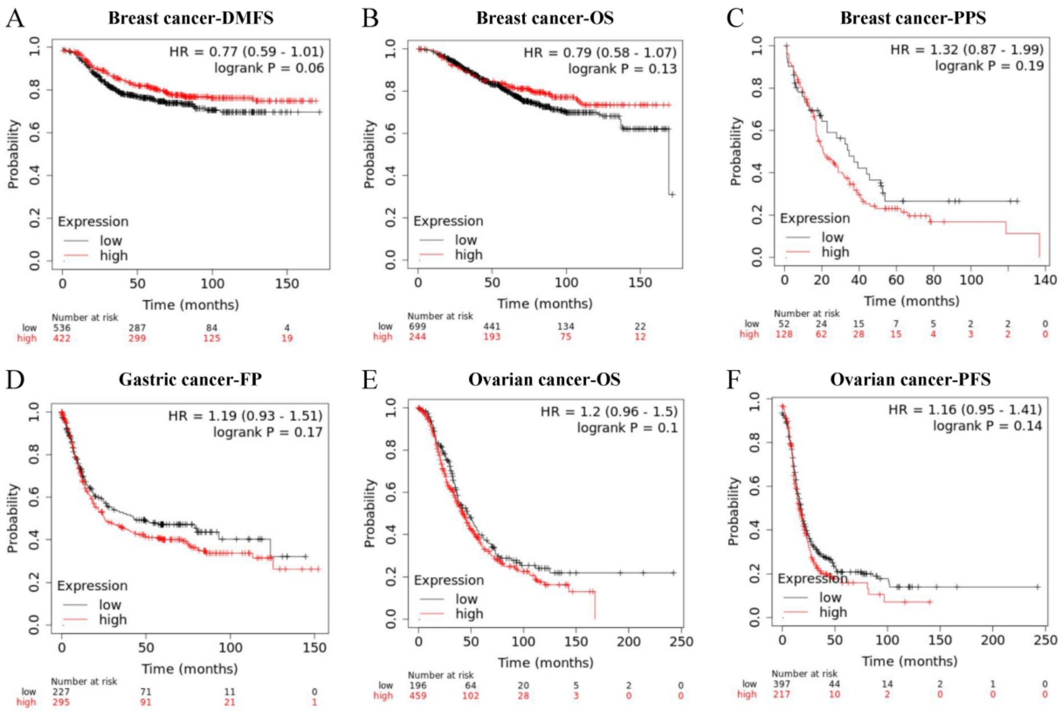

Figure S1. No correlation between REV1 expression and prognosis of various types of cancer (Kaplan-Meier Plotter database)

Table S1. Information of in silico algorithms

**In-silico algorithm**	**Website**
Illustrator for biological sequences (IBS)	http://ibs.biocuckoo.org/
cBioPortal	https://www.cbioportal.org/
NCBI dbSNP database	https://www.ncbi.nlm.nih.gov/snp/
SIFT	http://sift.jcvi.org/
PolyPhen-2	http://genetics.bwh.harvard.edu/pph2/
PANTHER	http://pantherdb.org/
SNPs&GO	https://snps.biofold.org/snps-and-go/
PROVEAN	http://provean.jcvi.org/protein_batch_submit.php?species=human
PredictSNP	https://loschmidt.chemi.muni.cz/predictsnp/
Mutation Taster2	http://www.mutationtaster.org/
Mutation assessor	http://mutationassessor.org/r3/
I-Mutant2.0	http://folding.biofold.org/i-mutant/i-mutant2.0.html
MUpro	http://mupro.proteomics.ics.uci.edu/
iStable	http://predictor.nchu.edu.tw/iStable/
Oncomine	https://www.oncomine.org/
TIMER	http://timer.cistrome.org/
PrognoScan	http://dna00.bio.kyutech.ac.jp/PrognoScan/index.html
Kaplan-Meier Plotter	https://kmplot.com/analysis/
Genomics of Drug Sensitivity in Cancer (GDSC)	https://www.cancerrxgene.org/
ArrayExpress	https://www.ebi.ac.uk/arrayexpress/

Table S2. REV1 expression in cancers vs. normal tissue in oncomine database

Cancer	Cancer type	*P*-value	Fold change	Rank	Sample	Reference (PMID)
Brain and CNS	Anaplastic Oligoastrocytoma	0.041	3.150	16 %	54	16204036
Breast	Invasive Breast Carcinoma Stroma	7.34E-24	-10.698	4 %	59	18438415
	Invasive Lobular Breast Carcinoma	0.029	-4.214	5 %	30	17389037
Kidney	Renal Wilms Tumor	0.011	2.132	12 %	67	19445733
Lymphoma	Anaplastic Large Cell Lymphoma, ALK-Negative	0.006	-2.045	8 %	64	19657361
Melanoma	Cutaneous Melanoma	0.001	2.598	3 %	87	18442402
Myeloma	Monoclonal Gammopathy of Undetermined Significance	1.18E-4	2.561	13 %	78	17023574
	Smoldering Myeloma	6.73E-5	3.207	18 %	78	17023574
Ovarian	Ovarian Carcinoma	3.67E-9	-2.709	4 %	195	18593951
Prostate	Benign Prostatic Hyperplasia Stroma	5.11E-4	2.890	1 %	101	17173048
Sarcoma	Leiomyosarcoma	5.71E-5	2.494	3 %	54	15994966
	Synovial Sarcoma	4.09E-4	2.212	7 %	54	15994966

Table S3. Relation between REV1 expression and patient prognosis of different cancer (Disease Free Survival) in Prognoscan database.

Cancer type	Dataset	N	P value	HR [95% CI]	Forest plot (DFS)
Breast cancer	GSE7378	54	0.609599	1.23 [0.55 - 2.76]	
	GSE4922-GPL96	249	0.871833	1.08 [0.44 - 2.64]	
	GSE4922-GPL97	249	0.332802	0.65 [0.27 - 1.56]	
	GSE4922-GPL97	249	0.693672	1.18 [0.51 - 2.74]	
	GSE4922-GPL97	249	0.0568602	0.75 [0.56 - 1.01]	
Colorectal cancer	GSE12945	51	0.683923	1.39 [0.28 - 6.88]	
	GSE17536	145	0.418046	1.75 [0.45 - 6.76]	
	GSE17536	145	0.822874	1.10 [0.47 - 2.56]	
	GSE17536	145	0.968807	1.02 [0.35 - 2.94]	
	GSE17536	145	0.47666	1.91 [0.32 - 11.27]	
	GSE14333	226	0.110955	0.79 [0.59 - 1.06]	
	GSE14333	226	0.519129	1.25 [0.64 - 2.43]	
	GSE14333	226	0.820051	0.96 [0.65 - 1.42]	
	GSE14333	226	0.776992	0.89 [0.38 - 2.06]	
	GSE17537	55	0.177586	2.67 [0.64 - 11.11]	
	GSE17537	55	0.816594	0.72 [0.05 - 11.30]	
	GSE17537	55	0.041971	2.72 [1.04 - 7.16]	
	GSE17537	55	0.352385	1.96 [0.47 - 8.13]	
Ovarian cancer	GSE26712	185	0.463136	0.91 [0.70 - 1.18]	

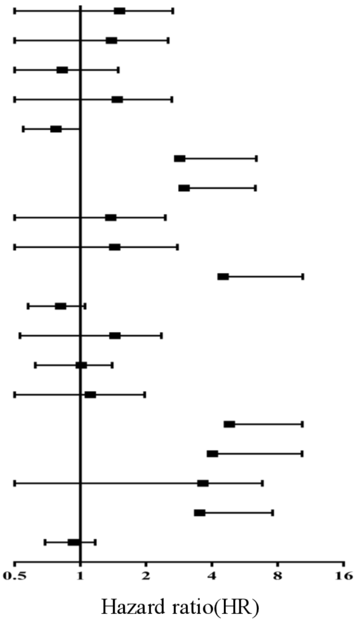

Table S4. Relation between REV1 expression and patient prognosis of different cancer (Disease Specific Survival) in Prognoscan database.

Cancer type	Dataset	N	P value	HR [95% CI]	Forest plot (DSS)
Bladder cancer	GSE13507	165	0.12466	0.72 [0.47 - 1.10]	
Blood cancer	GSE2658	559	0.186264	0.85 [0.66 - 1.08]	
	GSE2658	559	0.791105	1.04 [0.77 - 1.40]	
	GSE2658	559	0.240557	1.14 [0.91 - 1.43]	
	GSE2658	559	0.493749	0.78 [0.38 - 1.59]	
Breast cancer	GSE1456-GPL96	159	0.0988181	0.31 [0.07 - 1.25]	
	GSE1456-GPL97	159	0.844899	0.94 [0.50 - 1.77]	
	GSE1456-GPL97	159	0.569594	1.16 [0.69 - 1.96]	
	GSE1456-GPL97	159	0.10436	0.40 [0.13 - 1.21]	
	E-TABM-158	117	0.216211	1.54 [0.78 - 3.05]	
	GSE3494-GPL96	236	0.0638753	0.36 [0.12 - 1.06]	
	GSE3494-GPL97	236	0.991358	0.99 [0.32 - 3.07]	
	GSE3494-GPL97	236	0.954616	0.97 [0.33 - 2.82]	
	GSE3494-GPL97	236	0.00239758	0.56 [0.38 - 0.81]	
Colorectal cancer	GSE17536	177	0.11992	3.15 [0.74 - 13.36]	
	GSE17536	177	0.0399986	3.15 [1.05 - 9.39]	
	GSE17536	177	0.379168	1.36 [0.69 - 2.68]	
	GSE17536	177	0.118011	2.03 [0.84 - 4.92]	
	GSE17537	49	0.132844	3.15 [0.71 - 14.06]	
	GSE17537	49	0.834073	0.71 [0.03 - 18.10]	
	GSE17537	49	0.0960425	2.66 [0.84 - 8.43]	
	GSE17537	49	0.408214	2.24 [0.33 - 15.03]	
Lung cancer	GSE14814	90	0.498879	0.80 [0.43 - 1.52]	

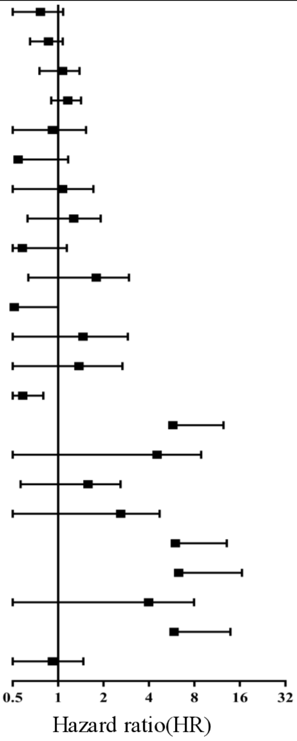

Table S5. Relation between REV1 expression and patient prognosis of different cancer (Distant Metastasis Free Survival) in Prognoscan database.

Cancer type	Dataset	N	P value	HR [95% CI]	Forest plot (DMFS)
Breast cancer	GSE19615	115	0.596085	1.48 [0.35 - 6.24]	
	GSE19615	115	0.950323	1.08 [0.09 - 13.81]	
	GSE19615	115	0.942283	1.04 [0.34 - 3.16]	
	GSE19615	115	0.407329	2.33 [0.31 - 17.29]	
	GSE6532-GPL570	87	0.842418	1.21 [0.19 - 7.73]	
	GSE6532-GPL570	87	0.972945	1.02 [0.34 - 3.02]	
	GSE6532-GPL570	87	0.689814	0.85 [0.37 - 1.93]	
	GSE6532-GPL570	87	0.865807	0.91 [0.31 - 2.65]	
	GSE9195	77	0.416972	0.51 [0.10 - 2.62]	
	GSE9195	77	0.818548	1.41 [0.07 - 26.97]	
	GSE9195	77	0.762963	1.37 [0.18 - 10.55]	
	GSE9195	77	0.47729	0.59 [0.14 - 2.52]	
	GSE12093	136	0.925703	0.93 [0.21 - 4.20]	
	GSE11121	200	0.126553	2.85 [0.74 - 10.89]	
	GSE2034	286	0.0635889	2.05 [0.96 - 4.35]	
	E-TABM-158	117	0.0586488	2.24 [0.97 - 5.16]	
	GSE2990	125	0.971598	1.01 [0.49 - 2.08]	
	GSE2990	54	0.333315	0.78 [0.48 - 1.29]	
	GSE7390	198	0.921475	0.97 [0.57 - 1.67]	
Eye cancer	GSE22138	63	0.0803999	51.26 [0.62 - 4232.50]	
	GSE22138	63	0.125544	1.49 [0.90 - 2.47]	
	GSE22138	63	0.792653	1.05 [0.71 - 1.56]	
	GSE22138	63	0.390537	1.24 [0.76 - 2.05]	

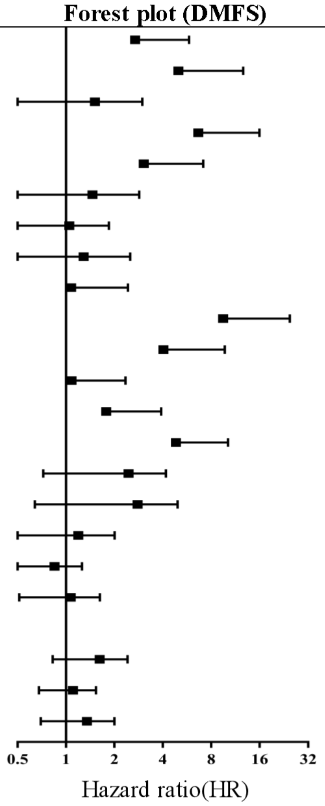

Table S6. Relation between REV1 expression and patient prognosis of different cancer (Overall Survival) in Prognoscan database.

Cancer type	Dataset	N	P value	HR [95% CI]
Bladder cancer	GSE5287	30	0.665197	0.85 [0.40 - 1.80]
	GSE13507	165	0.71569	0.94 [0.67 - 1.31]
Blood cancer	GSE12417-GPL96	163	0.496389	1.13 [0.80 - 1.60]
	GSE12417-GPL97	163	0.263211	0.47 [0.12 - 1.77]
	GSE12417-GPL97	163	0.0814435	1.70 [0.94 - 3.10]
	GSE12417-GPL97	163	0.290611	1.34 [0.78 - 2.31]
	GSE12417-GPL570	79	0.964967	0.98 [0.41 - 2.32]
	GSE12417-GPL570	79	0.257067	0.67 [0.34 - 1.34]
	GSE12417-GPL570	79	0.800187	1.08 [0.59 - 1.98]
	GSE12417-GPL570	79	0.493889	0.63 [0.16 - 2.40]
	GSE5122	58	0.121013	1.52 [0.90 - 2.56]
	GSE8970	34	0.316122	1.35 [0.75 - 2.45]
	GSE4475	158	0.0319974	0.51 [0.28 - 0.94]
	E-TABM-346	53	0.27852	0.47 [0.12 - 1.84]
	GSE16131-GPL96	180	0.589555	0.83 [0.43 - 1.62]
	GSE16131-GPL97	180	0.941426	0.99 [0.81 - 1.21]
	GSE16131-GPL97	180	0.493984	0.81 [0.45 - 1.47]
	GSE16131-GPL97	180	0.586029	1.20 [0.62 - 2.36]
Brain cancer	GSE4271-GPL96	77	0.0522892	2.12 [0.99 - 4.54]
	GSE4271-GPL97	77	0.129019	2.16 [0.80 - 5.86]
	GSE4271-GPL97	77	0.296463	1.48 [0.71 - 3.11]
	GSE4271-GPL97	77	0.695211	1.07 [0.77 - 1.47]
	GSE7696	70	0.874524	0.86 [0.13 - 5.64]
	GSE7696	70	0.972493	0.98 [0.24 - 4.03]
	GSE7696	70	0.780158	1.11 [0.54 - 2.25]
	GSE7696	70	0.32244	1.53 [0.66 - 3.55]
	GSE4412-GPL96	74	0.897872	0.92 [0.28 - 3.08]
	GSE4412-GPL97	74	0.950957	0.97 [0.38 - 2.47]
	GSE4412-GPL97	74	0.121916	0.75 [0.52 - 1.08]
	GSE4412-GPL97	74	0.371906	1.69 [0.53 - 5.34]
	GSE16581	67	0.105168	0.12 [0.01 - 1.56]
	GSE16581	67	0.277432	0.06 [0.00 - 10.10]
	GSE16581	67	0.791708	0.70 [0.05 - 10.11]
	GSE16581	67	0.0304626	0.08 [0.01 - 0.78]
Breast cancer	GSE1456-GPL96	159	0.553857	0.69 [0.21 - 2.33]
	GSE1456-GPL97	159	0.687437	0.91 [0.59 - 1.42]
	GSE1456-GPL97	159	0.124146	0.48 [0.19 - 1.22]
	GSE1456-GPL97	159	0.655339	1.15 [0.62 - 2.13]
	E-TABM-158	117	0.147334	1.53 [0.86 - 2.74]
	GSE7390	198	0.918312	1.03 [0.58 - 1.82]
Colorectal cancer	GSE12945	62	0.857137	0.91 [0.34 - 2.43]
	GSE17536	177	0.0469921	2.63 [1.01 - 6.85]
	GSE17536	177	0.481545	1.24 [0.68 - 2.24]
	GSE17536	177	0.0468463	2.19 [1.01 - 4.74]
	GSE17536	177	0.0690252	3.21 [0.91 - 11.31]
	GSE17537	55	0.232361	1.71 [0.71 - 4.16]
	GSE17537	55	0.421357	1.72 [0.46 - 6.48]
	GSE17537	55	0.447129	1.64 [0.46 - 5.87]
	GSE17537	55	0.860802	1.26 [0.10 - 16.41]
Esophagus cancer	GSE11595	34	0.445203	1.60 [0.48 - 5.35]
Lung cancer	jacob-00182-CANDF	82	0.72729	0.88 [0.42 - 1.82]
	jacob-00182-HLM	79	0.645639	0.83 [0.37 - 1.85]
	jacob-00182-MSK	104	0.0649923	0.37 [0.13 - 1.06]
	GSE13213	117	0.818215	1.06 [0.64 - 1.77]
	GSE13213	117	0.562526	0.86 [0.51 - 1.45]
	GSE31210	204	0.00721406	0.14 [0.03 - 0.58]
	GSE31210	204	0.00175978	0.54 [0.37 - 0.79]
	GSE31210	204	0.000192497	0.05 [0.01 - 0.24]
	GSE31210	204	0.000720138	0.25 [0.11 - 0.56]
	jacob-00182-UM	178	0.00216317	0.37 [0.20 - 0.70]
	GSE11117	41	0.856238	0.94 [0.50 - 1.79]
	GSE11117	41	0.264579	1.36 [0.79 - 2.31]
	GSE3141	111	0.690872	1.06 [0.79 - 1.43]
	GSE3141	111	0.528498	0.79 [0.39 - 1.63]
	GSE3141	111	0.381012	0.85 [0.59 - 1.22]
	GSE3141	111	0.102416	0.52 [0.23 - 1.14]
	GSE14814	90	0.397778	0.79 [0.45 - 1.37]
	GSE4716-GPL3694	50	0.0591493	0.40 [0.15 - 1.04]
	GSE4573	129	0.937666	0.97 [0.40 - 2.33]
	GSE17710	56	0.545164	0.80 [0.40 - 1.63]
	GSE17710	56	0.13197	0.59 [0.29 - 1.17]
	GSE17710	56	0.683345	0.89 [0.52 - 1.53]
Ovarian cancer	GSE9891	278	0.016573	1.72 [1.10 - 2.67]
	GSE9891	278	0.0191431	2.15 [1.13 - 4.08]
	GSE9891	278	0.0876177	1.51 [0.94 - 2.42]
	GSE9891	278	0.0221283	1.50 [1.06 - 2.12]
	DUKE-OC	133	0.219358	0.62 [0.29 - 1.33]
	GSE8841	81	0.31447	0.53 [0.15 - 1.82]
	GSE8841	81	0.00360352	4.88 [1.68 - 14.17]
	GSE26712	185	0.597626	0.93 [0.70 - 1.23]
	GSE17260	110	0.945674	1.02 [0.62 - 1.68]
	GSE17260	110	0.357439	0.76 [0.42 - 1.36]
	GSE14764	80	0.120711	0.41 [0.13 - 1.27]
Skin cancer	GSE19234	38	0.888652	0.92 [0.28 - 3.05]
	GSE19234	38	0.991068	1.00 [0.53 - 1.86]
	GSE19234	38	0.0776253	4.61 [0.84 - 25.17]
	GSE19234	38	0.356873	1.29 [0.75 - 2.20]

Forest plot (OS)

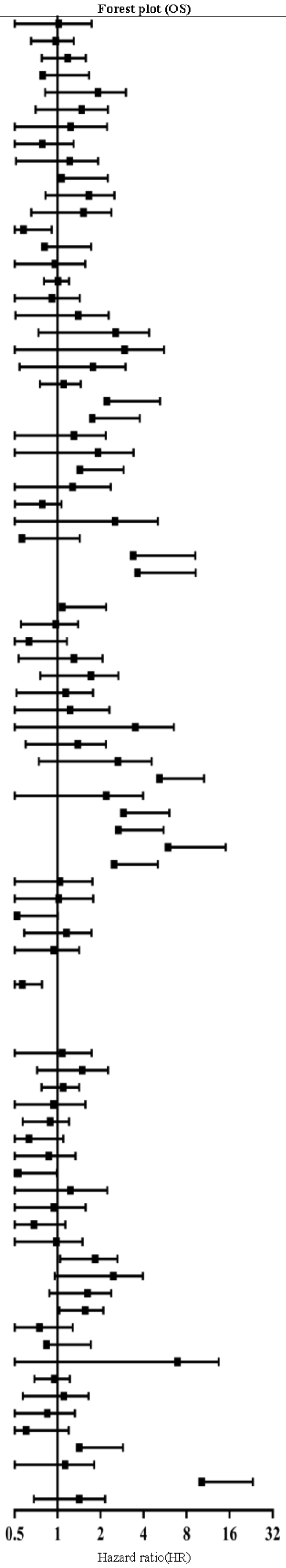

Table S7. Relation between REV1 expression and patient prognosis of different cancer (Relapse Free Survival) in Prognoscan database.

Cancer type	Dataset	N	P value	HR [95% CI]	Forest plot (RFS)
Breast cancer	GSE12276	204	0.0488286	1.52 [1.00 - 2.32]	
	GSE12276	204	0.19382	0.91 [0.80 - 1.05]	
	GSE12276	204	0.94301	0.98 [0.63 - 1.55]	
	GSE12276	204	0.930226	1.02 [0.72 - 1.44]	
	GSE6532-GPL570	87	0.972945	1.02 [0.34 - 3.02]	
	GSE6532-GPL570	87	0.689814	0.85 [0.37 - 1.93]	
	GSE6532-GPL570	87	0.865807	0.91 [0.31 - 2.65]	
	GSE6532-GPL570	87	0.842418	1.21 [0.19 - 7.73]	
	GSE9195	77	0.785471	1.43 [0.11 - 18.63]	
	GSE9195	77	0.814991	1.24 [0.20 - 7.53]	
	GSE9195	77	0.224012	0.45 [0.12 - 1.64]	
	GSE9195	77	0.188121	0.37 [0.09 - 1.62]	
	GSE1378	60	0.16519	0.64 [0.34 - 1.20]	
	GSE1379	60	0.0593989	0.54 [0.29 - 1.02]	
	GSE1456-GPL96	159	0.0524453	0.31 [0.10 - 1.01]	
	GSE1456-GPL97	159	0.825157	0.94 [0.55 - 1.61]	
	GSE1456-GPL97	159	0.232632	1.31 [0.84 - 2.05]	
	GSE1456-GPL97	159	0.0687392	0.41 [0.15 - 1.07]	
	E-TABM-158	117	0.147334	1.53 [0.86 - 2.74]	
	GSE2990	125	0.818676	1.07 [0.62 - 1.85]	
	GSE2990	62	0.572904	0.89 [0.60 - 1.33]	
	GSE7390	198	0.328144	1.25 [0.80 - 1.93]	
Head and neck cancer	GSE2837	28	0.882427	0.96 [0.55 - 1.67]	
	GSE2837	28	0.636922	1.51 [0.27 - 8.44]	
	GSE2837	28	0.207765	0.61 [0.29 - 1.31]	
	GSE2837	28	0.252531	0.41 [0.09 - 1.87]	
Lung cancer	GSE31210	204	0.0485166	0.35 [0.12 - 0.99]	
	GSE31210	204	0.0150541	0.68 [0.50 - 0.93]	
	GSE31210	204	3.33E-05	0.09 [0.03 - 0.28]	
	GSE31210	204	1.30E-05	0.26 [0.14 - 0.47]	
	GSE8894	138	0.0290771	0.00 [0.00 - 0.55]	
	GSE8894	138	0.270455	0.76 [0.46 - 1.24]	
	GSE8894	138	0.0771833	0.76 [0.57 - 1.03]	
	GSE8894	138	0.251725	0.78 [0.52 - 1.19]	
	GSE17710	56	0.0634709	0.54 [0.28 - 1.04]	
	GSE17710	56	0.505118	0.84 [0.51 - 1.39]	
	GSE17710	56	0.24526	0.67 [0.35 - 1.31]	

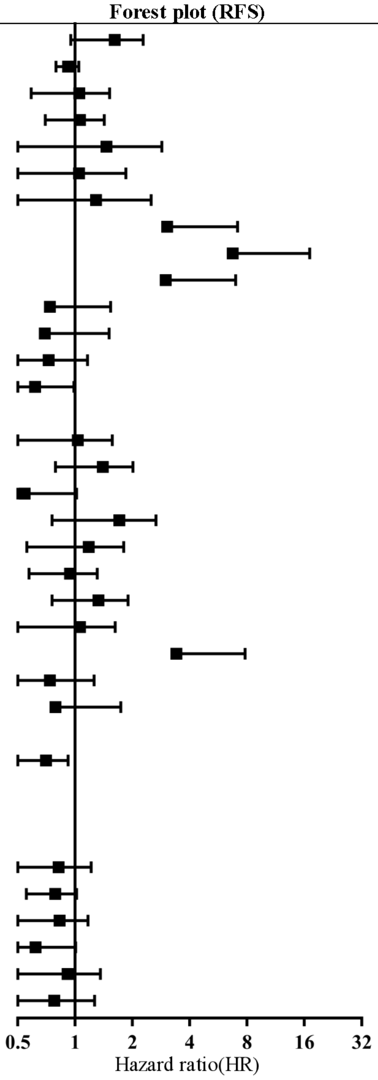

Table S8. The correlation between REV1 expression and drug pathway's IC50 value in different tumors.

TCGA Cancer[a]	Pathway Name	Correlation coefficient	P Value	Significant Codes[b]
ALL	Genome integrity	-0.099622518	0.047577029	*
ALL	Metabolism	-0.221896453	0.002994334	**
ALL	RTK signaling	0.070366236	0.025996342	*
CLL	Genome integrity	0.405973125	0.003442673	**
COREAD	DNA replication	-0.100298937	0.00866536	**
COREAD	Hormone-related	-0.185396031	0.006042912	**
COREAD	JNK and p38 signaling	-0.161438204	0.006125193	**
COREAD	Other, kinases	-0.067877099	0.004106506	**
COREAD	Protein stability and degradation	-0.150868204	0.009957252	**
COREAD	RTK signaling	-0.067158748	0.004668584	**
COREAD	WNT signaling	-0.135752484	0.007566221	**
DLBC	IGF1R signaling	0.211712913	0.027106539	*
ESCA	RTK signaling	-0.114712088	2.20E-05	***
HNSC	DNA replication	0.084217244	0.033296019	*
HNSC	ERK MAPK signaling	0.07661542	0.045173729	*
LAML	Cell cycle	0.090696845	0.036146889	*
LAML	Genome integrity	0.129079404	0.007024007	**
LAML	Hormone-related	0.213769529	0.015398123	*
LAML	Other, kinases	0.090785254	0.002202404	**
LAML	RTK signaling	0.080959529	0.006192631	**
LAML	WNT signaling	0.149075003	0.023748975	*
LCML	Other	0.094252767	0.044024138	*
LGG	Apoptosis regulation	0.213277621	0.002553999	**
LGG	Cell cycle	0.115418185	0.038152719	*
LGG	Chromatin histone methylation	0.294356308	0.016436081	*
LGG	DNA replication	0.144013879	0.018550047	*
LGG	JNK and p38 signaling	0.205238506	0.029943383	*
LGG	Other	0.091313425	0.010378774	*
LGG	Other, kinases	0.116202744	0.00167373	**
LGG	PI3K/MTOR signaling	0.086635449	0.037650125	*
LGG	RTK signaling	0.098392165	0.008287595	**
LIHC	ABL signaling	0.509178389	0.043963058	*
LUAD	Other, kinases	-0.044033807	0.036001892	*
LUAD	RTK signaling	-0.079226859	0.000155105	***
LUAD	WNT signaling	-0.09672753	0.029427361	*
MB	Cell cycle	-0.236764662	0.045239212	*
MB	Other, kinases	-0.239959285	0.002033995	**
MM	IGF1R signaling	0.32453541	0.011411441	*
MM	PI3K/MTOR signaling	0.097353093	0.023031369	*
MM	RTK signaling	0.104718798	0.006750413	**
NB	ERK MAPK signaling	0.092593665	0.041733349	*
NB	Hormone-related	0.228543073	0.011690825	*

NB	Other, kinases	0.076846482	0.008026697	**
NB	PI3K/MTOR signaling	0.073850165	0.023631069	*
OV	Other, kinases	0.06266377	0.036256985	*
OV	RTK signaling	0.082022932	0.005566808	**
OV	WNT signaling	0.133017661	0.031697367	*
SCLC	Cell cycle	0.14821341	2.37E-06	***
SCLC	Chromatin histone acetylation	0.072150006	0.047799355	*
SCLC	Cytoskeleton	0.134906114	0.004186677	**
SCLC	DNA replication	0.080560988	0.020812051	*
SCLC	ERK MAPK signaling	0.151804572	3.20E-06	***
SCLC	Hormone-related	0.123678127	0.049410753	*
SCLC	IGF1R signaling	0.193066922	0.007451844	**
SCLC	JNK and p38 signaling	0.150488741	0.005857	**
SCLC	Mitosis	0.111374776	0.001249168	**
SCLC	Other, kinases	0.1359607	1.26E-10	***
SCLC	PI3K/MTOR signaling	0.101445527	1.65E-05	***
SCLC	RTK signaling	0.110633374	1.63E-07	***
SCLC	WNT signaling	0.235371576	3.10E-07	***
SKCM	Genome integrity	-0.078554946	0.020929108	*
THCA	Cell cycle	0.140121636	0.040097086	*
THCA	ERK MAPK signaling	0.128847029	0.045697054	*
THCA	Hormone-related	0.367392381	0.002408781	**
THCA	JNK and p38 signaling	0.20789931	0.042095853	*
THCA	Other, kinases	0.150215444	0.000433588	***
THCA	PI3K/MTOR signaling	0.133279351	0.005260653	**
THCA	RTK signaling	0.12084359	0.005473799	**
UCEC	Chromatin histone methylation	-0.345072101	0.039288343	*

[a] TCGA Cancer: ALL: Acute lymphoblastic leukemia; BLCA: Bladder Urothelial Carcinoma; BRCA: Breast invasive carcinoma; CESC: Cervical squamous cell carcinoma and endocervical adenocarcinoma; CLL: Chronic Lymphocytic Leukemia; COREAD: Colon adenocarcinoma and Rectum adenocarcinoma; DLBC: Lymphoid Neoplasm Diffuse Large B-cell Lymphoma; ESCA: Esophageal carcinoma; GBM: Glioblastoma multiforme; HNSC: Head and Neck squamous cell carcinoma; KIRC: Kidney renal clear cell carcinoma; LAML: Acute Myeloid Leukemia; LCML: Chronic Myelogenous Leukemia; LGG: Brain Lower Grade Glioma; LIHC: Liver hepatocellular carcinoma; LUAD: Lung adenocarcinoma; LUSC: Lung squamous cell carcinoma; MB: Medulloblastoma; MESO: Mesothelioma; MM: Multiple Myeloma; NB: Neuroblastoma; OV: Ovarian serous cystadenocarcinoma; PAAD: Pancreatic adenocarcinoma; PRAD: Prostate adenocarcinoma; SCLC: Small Cell Lung Cancer; SKCM: Skin Cutaneous Melanoma; STAD: Stomach adenocarcinoma; THCA: Thyroid carcinoma; UCEC: Uterine Corpus Endometrial Carcinoma.

[b] P value Significant Codes: 0 ≤ *** < 0.001 ≤ ** < 0.01 ≤ * < 0.05
